# Local anesthesia for treatment of hernia in elder patients: Levobupicavaine or Bupivacaine?

**DOI:** 10.1186/1471-2482-13-S2-S30

**Published:** 2013-10-08

**Authors:** Rita Compagna, Gabriele Vigliotti, Tommaso Bianco, Maurizio Amato, Roberto Rossi, Francesca Fappiano, Antonello Accurso, Michele Danzi, Giovanni Aprea, Bruno Amato

**Affiliations:** 1 Department of Clinical Medicine and Surgery University of Naples Federico II Via S. Pansini, 5 - 80131 Napoli, Italy

## Abstract

**Background:**

Inguinal hernia is one of the most common diseases in the elderly. Treatment of this pathology is exclusively surgical and relies almost always on the use of local anesthesia. While in the past hernia surgery was carried out mainly by general anesthesia, in recent years there has been growing emphasis on the role of local anesthesia.

**Methods:**

The aim of our study was to compare intra-and postoperative analgesia obtained by the use of levobupivacaine to the same obtained by bupivacaine. Bupivacaine is one of the main local anesthetics used in the intervention of inguinal hernioplasty. Levobupivacaine is an enantiomer of racemic bupivacaine with less cardiotoxicity and neurotoxicity. The study was conducted from March 2011 to March 2013. We collected data of eighty patients, male and female, aged between 65 and 86 years, who underwent inguinal hernioplasty with local anesthesia.

**Results:**

Evaluation of intra-operatively pain shows that minimal pain is the same in both groups. Mild pain was more frequent in the group who used levobupivacaine. Moderate pain was slightly more frequent in the group who used bupivacaine. Only one reported intense pain. Two drugs seem to have the same effect at a distance of six, twelve, eighteen and twentyfour hours. Bupivacaine shows a significantly higher number of complications, as already demonstrated by previous studies. Degree of satisfaction expressed by patients has been the same in the two groups. Levobupivacaine group has shown a greater request for paracetamol while patients who experienced bupivacaine have showed a higher request of other analgesics.

**Conclusions:**

Clinical efficacy of levobupivacaine and racemic bupivacaine are actually similar, when used under local intervention of inguinal hernioplasty. In the field of ambulatorial surgery our working group prefers levobupivacaine for its fewer side effects and for its easy handling.

## Background

Inguinal hernia is one of the most common diseases in elderly. Treatment of this type of pathology is exclusively surgical and relies almost always on the contribution of local anesthesia. This type of anesthesia has significantly improved the treatment of inguinal hernia, significantly reducing recurrences, complications, recovery time and return to normal working activities. Hernia surgery should be approached according to a technique as simple and safe as possible that is at the same time accepted by the patient and easily realizable by the surgeon [1,2]. It is important find solutions which can be adapted to each individual patient, combining experience and new technologies. Surgery can be customized according to many parameters, as sex, age, comorbidity, lifestyle, type of hernia. Tailored Surgery is a sort of personalized surgery, individualized, built on the needs and characteristics of our patient. Concept of Tailored Surgery encompasses not only technical-surgical and prosthetic choices but also anesthetic (assisted local, spinal or loco-regional, general). According to recent guidelines of the European Hernia Society, published on "Hernia" in 2009, treatment of a hernia in primary election can always take advantage of local anesthesia. This is a grade A recommendation, with high scientific impact [3,4]. The simultaneous use of local anesthetic drugs with a long duration of action, but very powerful such as Levobupivacaine (Chirocaine), in addition to drugs equally potent, but duration of immediate action, such as Mepivacaine (Carbocaine), allow optimization of intra-and post-operatively anesthesia. Finally, do not forget that we are talking about local assisted anesthesia and therefore the contribution of the anesthetist, and the overall effectiveness of the anesthesia, are essential to ensure the maximum comfort to the patient intraoperatively [5]. This type of anaesthesia consists of several phases: the first, percutaneous, may be made without distinction by the surgeon or anesthesiologist, while the last phase, incisional, is exclusively of surgical pertinence, as it is the task of the surgeon to identify the points of landmarks, locate and infiltrate properly. Local Assisted Anesthesia by truncal block / incisional has several advantages: safety, even in patients at risk; effectiveness, commitment to anesthetic proportionate intervention, minimally invasive anesthetic technique, simple and reproducible. Currently local assisted anesthesia is the procedure of choice in primary unilateral inguinal hernias treated in election. There are no absolute contraindications to the anesthetic block. If anything, there are relative contraindications: poor patient, especially at a young age, morbid obesity, bilateral hernioplasty, bulky inguinal hernias [6]. Purpose of our study has been to compare clinical efficacy of two anesthetics, levobupivacaine and bupivacaine, commonly used during surgical treatment of inguinal hernia [23-25].

## Methods

From March 2011 to March 2013 we have studied eighty patients recovered in the department of General and geriatric Surgery, University of Naples "Federico II", affected by inguinal hernia.

Patients have been divided into two groups, corresponding to the two drugs that we studied. Patients have been interviewed at the end of the surgery. As benchmark for evaluation of pain we have used VAS scale. During the interview, we have focused attention on some particular aspects: intra-operative pain, post-operative pain, complications, need of analgesics in the postoperative period and the complessive satisfaction of patients. We have collected data of eighty patients, male and female, aged between 66 and 86 years, who underwent inguinal hernioplasty under local anesthesia.

In Table 1 we have reported main characteristics of our patients. Patients have been divided into two groups using a double-blind randomized system. The first group (A) received Levobupivacaine (n = 40), the second (B) received bupivacaine (n = 40). During surgery, the patients have been monitored with ECG intraoperative and pulse oximeter. In the first group twentytwo patients were treated for direct hernia and eighteen for indirect hernia. In the second group twentyone patients were treated for direct hernia and nineteen patients for indirect hernia. In Levobupivacaine group, the mean operative time was 43 minutes. While in the bupivacaine group the mean operative time has been 40 minutes exactly. In group A average anesthesiological time has been fiftyfive minutes. It has been fifty minutes for bupivacaine. Amount of fentanyl used has been respectively 115 mcg in the first group of interventions and 120 mcg in the second group. In the levobupivacaine group, the ratio right / left for the operated site was twentytwo to eighteen; in the bupivacaine group this ratio has been twentyfive to fifteen. It was also reported the ASA scale: twenty patients of the first group were classified in stage I and twenty patients in stage II. In the second group eighteen patients were classified in stage I and twentytwo patients in stage II. No patients in stage III. Anesthetic block was made employing the following protocol: first phase, percutaneous, allowed us to obtain a block on the troncular selective ilioinguinal and iliohypogastric nerves through a puncture performed two cm medial to the anterior superior iliac spine, lateral to the rectus muscle of abdomen. For this purpose it has been used 7-8 cc of Levobupivacaine (or Bupivacaine) 7.5%. The second phase, percutaneous, allows us to block genital branch of the genitofemoral nerve, through a puncture performed below inguinal ligament, lateral to pubic tubercle. It has been used 2-3 cc of levobupivacaine (or Bupivacaine) 7.5%. The third phase, percutaneous, has been completed by infiltration of the surgical incision, using a 22 gauge spinal needle employing 10 or 15 cc of Mepivacaine hydrochloride at 2%. Anesthetic block has been completed in the incisional phase by means of an open air infiltration, performed for each anatomical floor in the course of surgery, using Mepivacaine hydrochloride 2%. Four points requiring infiltration: end of the external oblique muscle (8, 10 cc), pubic tubercle (2 cc), medial and lateral pillar external inguinal orifice (2, 3 cc), orifice internal inguinal (2 3 cc) ; other locations in case of need or in large hernias can be: funiculus in sub-cremasterica; genitofemoral nerve in the sub-cremasterica and the hernial sac. With regard to the surgical techniques, patients affected by direct inguinal hernias have been treated with inguinal Lichtenstein hernioplasty. Patients affected by indirect inguinal hernia instead have been treated with Rutkow and Robbins hernioplasty. Immediately after the operation, patients have been interviewed to establish the extent of intra-and post-operative pain and the degree of satisfaction with surgery performed under local anesthesia. As we said before, it has been used for the interview VAS scale. VAS scale is a straight line with two ends corresponding to "no pain" and the worst possible pain (or the maximum that he experienced). It is a one dimensional tool that quantifies what patients subjectively perceive as pain or as a relief in all their physical, psychological and spiritual variables without distinguishing which of these components plays a greater role. Patient have received this question: "From one to ten, what level of pain you feel?". This scale presents many important characteristics: it has the advantage of being simple, is easily understood by most patients, can easily be repeated and is particularly useful for monitoring the acute course[22].

**Table 1:** Patient characteristics

Parameters	Levobupivacaine (n = 40)	Bupivacaine (n = 40)	*P *value
Medium Age (Max-Min)	76 (86-66)	74 (83-65)	0,82

Sex (M/F)	22/18	22/18	

Medium Weight (kg)	66	68	0,34

Direct hernias	22 (55%)	21 (52%)	0,51

Indirect hernias	18 (45%)	19 (48%)	

Site (R/L)	22/18	25/15	

Medium Operating time	43 (50-36)	40 (48-32)	0,25

Anesthesia time	55 (65-55)	50 (55-45)	0,30

ASA status			0,65

1	20 (50%)	18 (45%)	

2	20 (50%)	22 (55%)	

3	0	0	

Fentanyl (mcg)	115	120	0,33


## Results

Table 2 shows data related to intra-operative pain. We reported patient results. Level pain has been classified from one to ten. One and two have been considered minimal values. Three and four mild values. Five, six and seven moderate pain. Eight, nine and ten intense pain. In the group of patients who has received levobupivacaine, we identified four patients with minimal pain, eighteen mild pain, seventeen moderate pain and one intense pain. In the group of patients who has received bupivacaine, four have experienced minimal pain, seventeen mild, eleven moderate pain and no one intense pain. Then we have focused our point of view on post-operative pain. Degree of post-operative pain has been evaluated in three positions: supine, from supine to sitting, and during a short walk.

**Table 2:** Intra-operative pain (VAS scale)

Pain	Levobupivacaine	Bupivacaine	*P *value
1 (Minimal)	1 (2,5%)	2 (5%)	0,32

2 (Minimal)	3 (7,5%)	2 (5%)	

3 (Mild)	10 (25%)	9 (22,5%)	

4 (Mild)	8 (20%)	8 (20%)	

5 (Moderate)	7 (17,5%)	8 (20%)	

6 (Moderate)	5 (12,5%)	6 (15%)	

7 (Moderate)	5 (12,5%)	5 (12,5%)	

8 (Intense)	1 (2,5%)	0 (0 %)	

9 (Intense)	0 (0 %)	0 (0 %)	

10 (Intense)	0 (0 %)	0 (0 %)	


Table 3 reports results of post-operative pain. In levobupivacaine group four patients have experienced pain in the supine position, six seated and four standing. In Bupivacaine group, four patients identified pain in supine position, six seated and six standing. Later we have collected impressions of patients even after several hours from surgery. Six hours after operation, six patients in the first group and six patients in the second group identified pain. Twelve hours after surgery, five patients in the first group and four in the second identified pain. Eighteen hours after surgery, three patients in the first group and three in the second referred pain. Twentyfour hours after surgery, two patient in the first group and two patient in the second group identified pain.

**Table 3:** Post-operative pain

Position	Levobupivacaine	Bupivacaine	*P *value
Supine	4 (10 %)	4 (10 %)	0,7

Sitting	6 (15 %)	6 (15 %)	0,9

Standing	4 (10 %)	6 (15 %)	0,3

Time after operation	Levobupivacaine	Bupivacaine	*P *value

6 h	6 (15 %)	6 (15 %)	0,75

12 h	5 (12,5 %)	4 (10 %)	0,4

18 h	3 (7,5 %)	3 (7,5 %)	0,12

24 h	2 (5%)	2 (5 %)	0,25


In Table 4 we have evaluated complications of threatment. In the first group four patients experienced nausea, four vomiting, two itching, and one infection. Instead in the bupivacaine group, five patients experienced nausea, five vomiting, one itching and two infection.

**Table 4:** Post-operative complications

Complications	Levobupivacaine	Bupivacaine	*P *value
Nausea	4 (10 %)	5 (12,5 %)	0,67

Vomiting	4 (10 %)	5 (12,5 %)	

Infection	1 (2,5 %)	1 (2,5 %)	

Itching	2 (5 %)	2 (5 %)	


Table 5 shows results about overall satisfaction. It was assessed using a scale of five levels. In group A of levobupivacaine, twentysix patients said they have been absolutely satisfied, six very satisfied, six moderately satisfied, two satisfied and no one has been disappointed. In the second group, twentyfive patients have been absolutely satisfied, seven have been very satisfied, five have been, three satisfied moderately satisfied, no one disappointed.

**Table 5:** Degree of patient satisfaction

Patient satisfaction	Levobupivacaine	Bupivacaine	*P *value
Absolutely satisfied	26 (65 %)	25 (62,5 %)	0,71

Very satisfied	6 (15 %)	7 (17,5 %)	

Moderately satisfied	6 (15 %)	5 (12,5 %)	

Satisfied	2 (5 %)	3 (7,5 %)	

Disappointed	0 (0 %)	0 (0 %)	


Table 6 collected data about need of analgesic during immediate post-operative phase. Patients of the first group who required paracetamol have been twentyeight. Twentyfour patients in the second group. Patients of first group who required other analgesics for pain relief within twenty-four hours were twelve. Sixteen patients of the second group required others analgesics.

**Table 6:** Need for analgesic

Analgesics	Levobupivacaine	Bupivacaine	*P *value
Request of paracetamol	28 (70 %)	24 (60 %)	0,85

Other analgesics	12 (30%)	16 (40 %)	0,44


## Statistical analysis

In this study, continuous variable was reported as an average, more or less the standard deviation, and analyzed using ANOVA (analysis of variance). ANOVA is a parametric test used in statistics to compute variance between two or more different groups. Analysis of variance is a set of statistical techniques that are part of the inferential statistics that allow us to compare two or more groups of data comparing the internal variability of these groups with the variability between groups. Categorical variables have been reported as proportions and analyzed using chi-square test. Chi-square test adopts chi-square variable causal to verify if null hypothesis is compatible with data. Values relating to intra-operative pain and post-operative pain, as well as those relating to the taking of analgesics during the postoperative course, have been always reported and analyzed through chi-square test. A P value less than 0.05 has been considered statistically significant. Based on previous studies, the difference in the level of pain between the group of levobupivacaine and bupivacaine was 1.5.

## Discussion

International literature shows how local anesthesia has more advantages compared to other kind of anaesthesia. A potential advantage of local anesthesia realized without any monitoring or additional drugs administered intravenously (the so-called local anesthesia not monitored) [7].

Levobupivacaine is a local anesthetic with long duration of action. It blocks nerve conduction of sensory and motor nerves, interacting predominantly with the voltage-gated sodium channels in the membrane of the cell, but also blocking potassium and calcium channels. Levobupivacaine also interferes with the transmission of the pulse and the conduction in other tissues where the effects on the central nervous system and cardiovascular system are the most important for the occurrence of clinical adverse reactions. Chirocaine is a compound based levobupivacaine hydrochloride. It is capable of producing a block on both the sympathetic system and on the parasympathetic system demonstrating hemodynamic changes significantly milder than Ropivacaine, which instead has the greatest influence on the sympathetic system with respect to that parasympathetic [8]. The dose of levobupivacaine is expressed as a basis, unlike the racemic Bupivacaine where the dose is expressed as a hydrochloride salt. This roughly translates into a 13% more active ingredient in the solutions of levobupivacaine compared to those of bupivacaine. As regards to the pharmacokinetic properties, in human trials, the kinetics of distribution of levobupivacaine after intravenous administration are essentially the same as bupivacaine. The plasma concentration of levobupivacaine following therapeutic administration depends on the dose and, as absorption from the site of administration is influenced by the vascularity of the tissue, the route of administration. It is available in two formulations: Vial of 10 ml polypropylene, in pack sizes of 5, 10 and 20 units, polipropilene vial of 10 ml in sterile blister packs of 5, 10 and 20 units. Chirocaine can be worked in a very large number of surgical procedures, can be administered in major surgery for epidural, intrathecal, in nerve conduction block device, in minor surgery for local infiltration and for ophthalmic use in order to obtain a peribulbar block. It could be used in the treatment of pain, as an analgesic in the course of delivery, both for bolus infusion, and also for the post-operative pain.

Among the uses of Chirocaine there are scientifically proven mastopexy interventions [9]. Local anesthesia applied during endarterectomy surgery allows the surgeon to assess the levels of cerebral perfusion in an awake patient, giving a better chance of cerebral protection during arterial clamping. All these elements indicate that such interventions performed under local anesthesia with levobupivacaine compounds offer greater chances of success with significantly reduced rates of morbidity and mortality [12-14]. Levobupivacaine is more effective to obtain analgesia with local infiltration compared to Ropivacaine, providing analgesia for postoperative period. Interventions of septoplasty and rhinoseptoplasty with an infiltration of levobupivacaine at 0.25% in the nasal region improve the post-operative analgesia and reduce the demand for additional analgesia during the twenty-four hours following nasal surgery. The post-operative analgesia achieved through the local infiltration of levobupivacaine has been demonstrated to be significantly more powerful and showed longer duration compared to the association lidocaine plus epinephrine. The same holds with regard to the interventions of mini-abdominoplasty [5]. In this case levobupivacaine has proved to be more effective and with a duration indeed higher than ropivacaine. Levobupivacaine can be the agent of first choice in the thoracic epidural block [10] , compared to the use of a Ropivacaine dose equivalent. It has also proved effective even in the interventions of arthroscopy and Carotid Endarterectomy [11][14]. Locally hernioplasty has proved to be the method with the minor impact on the functioning of organs and systems, as it appears to be safe, effective, with a low incidence of side effects, enabling a rapid mobilization of the patient and significantly reducing the time of hospitalization, in less than twenty-four hours [15]. Among rare complications of surgery, hernioplasty under local anesthesia include: cardiovascular instability, nausea, vomiting, urinary retention, scrotal hematoma, edema, infection, orchitis, testicular atrophy and recurrences. This kind of surgery shows a lower incidence of complications than same operation performed with general anesthesia. Compared with other types of anesthesia, post-operative complications of the respiratory and circulatory systems are significantly reduced [16]. The use of local anesthesia also allows the patient to be awake, aware, and thus able to collaborate actively conducting a stress-test by performing the Valsalva maneuver or a cough, which allows the surgeon to evaluate intra-operatively the presence of defects, latent trusses and sealing of the repair of plastic, reducing significantly the proportion of surgical failures [17,18]. The anesthetic block consists of four phases: First phase, percutaneous, provides the block troncular selective ilioinguinal nerves and iliohypogastric. Second phase, percutaneous, blocks the genital branch of the genitofemoral nerve, through a puncture performed below the inguinal ligament, lateral to the pubic tubercle. The third phase, percutaneous, provides for the infiltration of the surgical incision using a 22 gauge spinal needle. Anesthetic block is completed in the incisional phase by means of an open infiltration performed in each anatomical floor during the course of surgery [19]. Local anesthesia with levobupivacaine and bupivacaine is now a established and safe procedure with risks considerably reduced, a quick and full recovery of the patient's general condition and an immediate return to normal working activities. Data from the international literature indicate how the levobupivacaine is less toxic compared to bupivacaine, both at the cardiac level and at the neurological level [20,21]. The purpose of this study was to compare the perception of intra and post-operative pain, found as a result of intervention with the Levobupivacaine, compared to the same intervention carried out with racemic bupivacaine. We have used same dose for both anesthetics. Eight patients have been studied, have been randomly distributed in two groups, and have been classified on the basis of a number of variables: age, weight, sex, type of hernia, ASA Stadium and location of the hernia. First point on which we have focused our attention was intra-operative pain.

In the group of patients treated with levobupivacaine, 2,5 % of patients reported degree one of pain. 7,5 % degree two, 25 % degree three, 20 % degree four, 17,5 % degree five, 12,5 % degree six, 12,5 % degree seven, 2,5 % degree eight, 0% degree nine and ten. Degree one and two have been considered minimal pain, three and four mild pain, five, six and seven moderate pain and finally eight, nine and ten intense pain. So in the first group 10 % of patients has shown minimal pain, 45 % mild pain, 42,5 % moderate pain and 2,5 % intense pain. In the group of patients treated with bupivacaine, 5 % reported degree one of pain, 5 % degree two, 22,5 % degree three, 20 % degree four, 20 % degree five, 15 % degree six, 12,5 % degree seven, 0 % degree eight, nine and ten. Considering the same classification adopted for levoupivacaine, we can observe that 10% identified minimal pain, 42,5% mild pain, 47 % moderate pain and 0 % intense pain. Comparing two different groups we can say that minimal pain is the same for levoupivacaine and bupivacaine. Mild pain is greater in the first group. Moderate pain is stronger in the second group. Only one patient showed intense pain, in group of levobupivacaine.

Second point, post-operative pain. It has been assessed in three positions within twentyfour hours. In the first group, 15% of patients reported pain in supine position, 15% in sitting position and 10% standing up. In the second group, 10 % reported pain in the supine position, 15% in the sitting position and 15% standing up. Therefore data show same results for the first two positions and a slight preference for levobupivacaine in the upright position. With regard to the assessment of pain during twentyfour hours, we evaluated the impressions of the patient's at four time intervals: six, twelve, eighteen and twenty-four hours. In the levobupivacaine group, 15% of patients expressed pain relief after six hours, 12,5 % after 12 hours, 7,5 % after 18, 5 % after 24 h. In the bupivacaine group, 15 % of patients experienced pain after 6 h, 10 % after 12 h, 7,5 % after 18 h and 5 % after 24 It is therefore evident how Bupivacaine is preferred slightly after 12 h, while the two drugs appear to be equivalent at a distance of 6, 12 and 24 h.

Third point, postoperative complications. In Levobupivacaine group, 20% experienced symptoms such as nausea and / or vomiting, 5% itching, 2,5 % infection. In bupivacaine group, 25% noted nausea and / or vomiting, 5% itching, 2,5% infection. Bupivacaine shows a significantly higher number of complications, as already demonstrated by previous studies.

Fourth point, overall satisfaction. Patients that received levobupivacaine have been absolutely satisfied for 65 %. 15% very satisfied. 15 % moderately satisfied. 5 % satisfied and 0 % disappointed. Instead patients that received bupivacaine expressed 62,5% complete satisfaction, 17,5 % have been very satisfied, 12,5 % moderately satisfied, 7,5 % satisfied and 0 % disappointed. In neither of the two groups we have found signs of toxicity by local anesthetic, such as tinnitus, pallor circumorale, cardiovascular or neurological manifestations. So we can observe how degree of satisfaction has been the same for the two groups.

Finally, the last point on which we have focused our work has been need of analgesic during post-operative period. Seventy percent of patients who have received levobupivacaine required at least an analgesic (paracetamol) within twenty-four hours surgery and 30% required others analgesics. In the bupivacaine group, 60% took some paracetamol after twenty-four hours, 40 % required other analgesics. The request for paracetamol has been slightly higher in patients receiving levobupivacaine while request for other analgesic has been greater in group of bupivacaine.

## Conclusions

We can say that clinical efficacy of levobupivacaine and racemic bupivacaine are actually similar. When we perform inguinal hernioplasty surgery with local anaesthesia, Levobupivacaine could be preferred because it has a lower cardiac and neurological toxicity compared to bupivacaine, as previously demonstrated by other clinical studies. With a growing assertion of outpatient surgery, is clear and evident as levobupivacaine will can find more and more space in the future, in common clinical practice, because it has fewer side effects and better handling.

## Competing interests

The authors declare that they have no competing interests.

## Authors' contributions

RC: conception and design, interpretation of data, given final approval of the version to be published.

GV, TB, MA, RR, FF, AA, MD, GA: acquisition of data, drafting the manuscript, given final approval of the version to be published.

BA: conception and design, given final approval of the version to be published

## Authors' information

RC: Post-Graduate Doctorate in Vascular Surgery at University "Federico II" of Naples.

GV, TB, MA, RR, FF: Resident in General Surgery Training Programme at University "Federico II" of Naples.

AA, MD, GA: Aggregate Professor of Surgery at University "Federico II" of Naples, Italy.

BA: Associate Professor of Surgery at University "Federico II" of Naples, Italy.

## Declarations

Funding for this article has come from the author's personal funds.

This article has been published as part of *BMC Surgery *Volume 13 Supplement 2, 2013: Proceedings from the 26th National Congress of the Italian Society of Geriatric Surgery. The full contents of the supplement are available online at http://www.biomedcentral.com/bmcsurg/supplements/13/S2
